# Urinary and fecal potassium excretion prediction in dairy cattle: A meta-analytic approach

**DOI:** 10.3168/jdsc.2023-0440

**Published:** 2024-02-29

**Authors:** Joyce L. Marumo, P. Andrew LaPierre, Michael E. Van Amburgh

**Affiliations:** Department of Animal Science, Cornell University, Ithaca, NY 14853

## Abstract

Quantification of potassium (K) excretion in dairy cattle is important to understand the environmental impact of dairy farming. To improve and monitor the environmental impact of dairy cows, there is a need for a simple, inexpensive, and less laborious method to quantify K excretion on dairy farms. The adoption of empirical mathematical models has been shown to be a promising tool to address this issue. Thus, the current study aimed to develop empirical predictive models for K excretion in dairy cattle from urine and feces that can help evaluate efficiency and monitor the environmental impact of milk production. To develop urine K (K_Ur_, g/d) and fecal K (K_Fa_, g/d) excretion prediction models, published literature that involved 45 and 54 treatment means from 10 and 14 studies, respectively, were used. Some studies reported either urinary or fecal K excretion or both, but in total, treatment means used to develop the models were from 17 studies. The linear mixed models were fitted with the fixed effect of K intake, DMI, dietary K content, urine volume, milk yield, and water intake, and the random effect of study weighted according to the number of observations. Leave-one-study out cross-validation was used to evaluate the performance of the proposed models and the best model was based on the lowest root mean square prediction error as a percentage of the observed mean values (RMSPE%) and highest concordance correlation coefficient (CCC). As expected, most daily K excretion was through urine (202.5 ± 92.1 g/d) than through feces (43.5 ± 21.0 g/d), and among the proposed models, the model including dietary K concentration showed poor predictive ability for both K_Ur_ and K_Fa_ with the lowest CCC values (−0.15 and −0.02, respectively) and systematic bias. The model developed using DMI to predict K_Fa_ excretion showed reasonable accuracy, as indicated by RMSPE, CCC, and R^2^_marginal_ of 46.6%, 0.42, and 48%, respectively. Among the proposed models for K_Ur_ and K_Fa_, the model with K intake demonstrated better predictive performance, showing minimal systematic bias and random errors due to data variability of >92%. While these proposed models suggested that reducing K intake can lead to a decrease in K excretion, it is important to ensure that dairy cows receive adequate amounts of this nutrient to maintain optimal health and productivity, especially during periods of heat stress.

## Highlights

• Overall, the daily K excreted via urine was greater than through feces in dairy cattle.
• This study developed simple linear mixed models for the prediction of K_Ur_ and K_Fa_ excretion in dairy cattle.
• Among all the proposed models, K_Ur_ and K_Fa_ were best predicted with K intake with minimal systematic biases.

## Graphical Abstract

**Figure:**
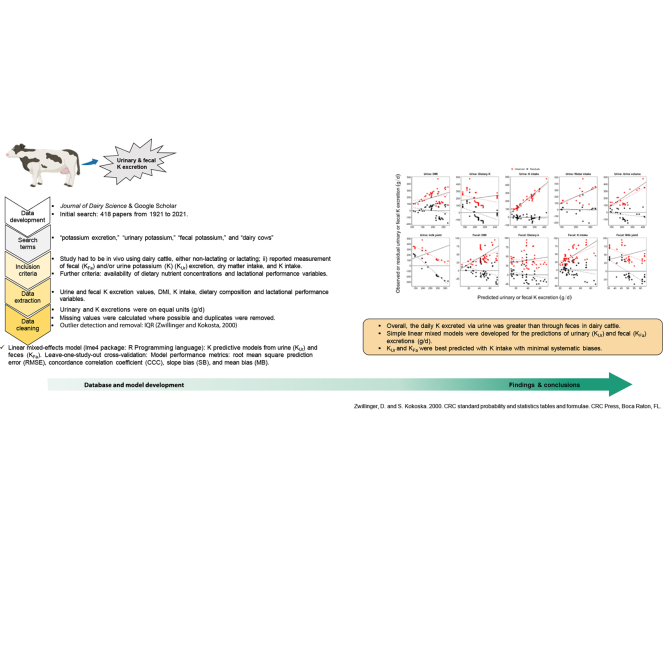
**Summary:** Understanding potassium (K) excretion in dairy cattle is vital for assessing the environmental impact of producing milk. To enhance the environmental monitoring of dairy cattle, there is a need for a simple, costeffective, and less labor-intensive method to quantify K excretion on dairy farms. Therefore, developing empirical predictive models for K excretion in dairy cattle would provide a more efficient and cost-effective way to quantify K excretion. Models to predict urinary (K_Ur_, g/day) and fecal K (K_Fa_, g/day) excretion were constructed using published literature from various studies. Urinary and fecal K excretion regression models showed better predictive performance with minimal systematic biases.

Producing milk is a vital component of agriculture and an important contributor to the global food supply. However, the environmental impact of dairy farming has become an increasing concern due to the potential negative effects on air, water, and soil quality (Baskaran et al., 2009). One such contributing factor is the excretion of excessive nutrients, including potassium (K). Potassium is the third-most abundant macromineral for dairy cows that must be supplied daily due to its limited body storage and high demand for biological processes (NASEM, 2021). According to the NRC (2001), the minimum dietary K recommendation for dairy cows ranges from 0.55% to 1.0% on a DM basis, with no substantial change in the latest report by NASEM (2021). Proper K feeding according to the cow's requirements ensures vital functions such as maintenance of osmotic volume regulation, nerve impulse transmission, water balance, and muscle contraction (NASEM, 2021). Earlier research pointed out that under heat stress conditions, dairy cows increase salivation, panting, and sweating rates to maintain normal body temperature leading to faster excretion of K than they consume in the diet (West et al., 2003). Early lactation cows have been noted to be in a negative K balance due to reduced DMI coupled with greater K excretion and greater milk K secretion (Nennich et al., 2005). Thus, an increase in K supplementation in the cows' diet has been linked to the alleviation of heat stress and an increase in daily milk yield (Schneider et al., 1986; West et al., 1991; Silanikove et al., 1997). However, excessive K intake can result in significant excretion in urine and feces, primarily through urine (Lomba et al., 1969), posing environmental implications, including soil nutrient imbalance, water pollution, and negative impacts on plant growth (Xu et al., 2020). Additionally, excessive accumulation of K in soils from manure application increases the risk of magnesium deficiency in dry and transition cattle through feeding forage that has been overfertilized with K from manure (NRC, 2001).

Quantifying K excretion using practical approaches is essential for assessing and managing its environmental impact. An in vivo nutrient balance measurement is essential for evaluating mineral utilization (Faulkner and Weiss, 2017), environmental impact (Tomlinson et al., 1996), and dietary needs (NRC, 2001). This approach requires quantification of excreta, which involves collecting urine and fecal samples, and the total collection of these samples can be challenging and impractical under certain farm conditions (Tebbe and Weiss, 2018). In addition, quantification of concentration of this mineral can be done using laboratory techniques such as spectrometry, and assays, which can be costly and time consuming. Therefore, simpler and less expensive approaches such as the use of predictive models to quantify mineral excretion from dairy cattle are needed. Previous publications have developed predictive models for other excreted nutrients, including nitrogen (Higgs et al., 2012; Johnson et al., 2016) and phosphorus (Wu et al., 2000; Alvarez-Fuentes et al., 2016); however, there are very limited studies that predicted K excretion by dairy cows (Kojima et al., 2005). A recent study by Plöntzke et al. (2022) developed kinetic models to understand the dynamics of K excretion in nonlactating and lactating dairy cows. Kojima et al. (2005) developed empirical prediction models for urinary and fecal K excretion in lactating and dry cows, but they did not find a significant predictor for fecal K excretion in dry cows. Furthermore, Bannink et al. (1999) established prediction models based on the relationship between the intake of apparently digestible K and its secretion in milk and urine. However, these measurements can be challenging to obtain or use on a farm basis as they require in vivo digestibility trials (Nennich et al., 2006).

Thus, the existing body of literature could potentially be used to identify important variables needed to develop predictive equations for K excretion. This research aims to develop empirical models that predict urinary and fecal K excretion in dairy cattle and evaluate their performances.

A literature search was conducted in the *Journal of Dairy Science* (**JDS**) (https://www.journalofdairyscience.org/) and Google Scholar. This resulted in papers from 1921 through 2021 using the search terms “potassium excretion,” “urinary potassium,” “fecal potassium,” and “dairy cows.” The initial search in JDS and Google Scholar resulted in a total of 418 papers related to K_Ur_ or K_Fa_ excretion in dairy cows.

The studies included in the database had to meet 2 criteria: (1) the study had to be in vivo using dairy cattle, either nonlactating or lactating; and (2) report either fecal or urine excretion, DMI, and K intake. Further criteria included the availability of dietary nutrient concentrations and milk yield. The K_Ur_ and K_Fa_ measurements were converted into grams per day. In the case where K intake was not reported, it was calculated using DMI and dietary K. The summary and descriptive statistics for the variables considered in the databases are in Table 1. Following the first screening process, 49 abstracts, 20 reviews, 15 interpretive summaries, 29 unrelated papers, and 1 duplicate were excluded from our database. An additional search conducted on Google Scholar resulted in 14 papers. The papers were included in the screening process, ultimately leading to a final selection of 17 papers that met the established inclusion criteria. These selected papers contributed a total of 45 and 54 treatment means in the urinary and fecal K databases, respectively. The final urinary K database included 37 observations for lactating cows and 8 for nonlactating cows from 9 papers and 4 papers, respectively. The fecal K database comprised 54 observations, with 46 from lactating cows (12 papers) and 8 (4 papers) from nonlactating cows. Due to limited observations in both subsets, we were unable to build separate predictive K_Ur_ and K_Fa_ models for lactating and nonlactating cows. No differences were observed in the relationship between the dependent variables and the predictor variables when the lactating cows-only subset was used. Therefore, the final databases for the urinary and fecal databases comprised both lactating and nonlactating cows.

**Table 1:** Summary descriptive statistics of the databases used to develop the predictive models from urine and fecal studies^1^, ^2^

Item	n	Mean	SD	Min	Max
Urinary database					
Urine K, g/d	45	202.5	92.1	70.2	476.0
DMI, kg/d	45	16.9	5.4	4.4	27.6
K intake, g/d	45	316.6	144.3	105.3	686.0
Dietary K, % of DM	45	1.7	0.66	0.79	2.73
Water intake, kg/d	18	83.6	44.2	20.9	146.6
Urine volume, kg/d	26	21.1	8.02	10.5	39.9
Milk yield, kg/d	20	31.2	6.20	21.6	41.5
Fecal database					
Fecal K, g/d	54	43.5	21.0	15.5	94.7
DMI, kg/d	54	17.9	5.8	4.4	27.6
K intake, g/d	54	282.0	125.8	105.3	583.0
Dietary K, % of DM	49	1.5	0.50	0.79	2.46
Milk yield, kg/d	37	30.0	7.80	20.1	41.6

1 West et al., 1987; Fisher et al., 1994; Rahnema et al., 1994; Delaquis and Block, 1995; Tomlinson et al., 1996; Khorasani et al., 1997; Silanikove et al., 1997; James et al., 1999; Kume et al., 2001, 2004; Kojima et al., 2005; Charbonneau et al., 2008; Rérat et al., 2009; Harrison et al., 2012; Eriksson and Rustas, 2014; Tebbe and Weiss, 2018; Tebbe et al., 2018.

2 n = number of treatment means used to develop the proposed models; Min = minimum; Max = maximum.

All urinary and fecal databases were screened for outliers using variables such as fecal or urine K excreted (g/d), DMI, K intake, and urine volume. Outliers were removed using the interquartile range method (Zwillinger and Kokoska, 2000) with a factor of 1.5 indicating extreme values. One treatment mean from the study of Eriksson and Rustas (2014) was identified as an outlier in the fecal database and excluded from the final analysis. However, the same study was kept in the urine database because the models were developed with and without these data and were shown to have had no influence on the model prediction performances. One study (Delaquis and Block, 1995) was excluded in the development of the urinary K excretion model due to unrealistically low urinary K excretion (<27 g/d) in the treatment groups with a K intake of >320 g/d. In addition, the study of Kume et al. (2008) was excluded from the analysis in the urine database because the study used data from the studies (Kume et al., 2004; Kojima et al., 2005) that are also part of the databases in the current study.

Data analysis was performed in R (R Core Team, 2022) within RStudio (Posit team, 2023). The linear mixed effect predictive models were fitted using the lme4 package (Bates et al., 2015) to predict K_Ur_ or K_Fa_ excretion with the random effect of study and fixed effect of the explanatory variables, and the model was given as

[1] *Y* = *β*_0_ + *β*_1_*X*_1_ + *S_i_* + *e_ij_*,

where *Y* denotes the dependent variable of urinary or fecal K excretion (g/d), *β*_0_ denotes the fixed effect of the intercept, *X*_1_ denotes the fixed effects of the independent variable (DMI [kg/d], K intake [g/d], dietary K content [% of DM], water intake [kg/d], milk yield [kg/d], and urine volume [kg/d]), and *β*_1_ denotes the corresponding slope, *S_i_* denotes the random effect of the studies, and *e_ij_* denotes the random error. To account for the accuracy of the reported treatment means, the simple linear mixed models were weighted by the number of observations with the WEIGHT statement specified in the lmer function (St-Pierre, 2001).

To evaluate the prediction accuracy of the developed models, leave-one-out cross-validation (**LOOCV**) was performed with study regarded as a fold, whereby one study was used as the validation data and the remaining studies were used as the training data in each iteration. The model performance metrics were then determined using the predictions resulting from the LOOCV procedure (Table 2). The R-squared marginal was calculated using MuMIN package (Bartoń, 2022).

**Table 2:** Potassium excretion (g/d) prediction model equations and model performance evaluations using urine and fecal databases^1^

Proposed model^2^	n	Model performance						R^2^_marginal_
		RMSPE, %	MB, %	SB, %	ED, %	RSR	CCC	
K_Ur_ (g/cow per day)								
= 70.05 (±39.10) +7.86(±2.01) × DMI	45	44.6	0.00	4.55	95.45	0.98	0.19	0.28
= 1.08 (±9.58) + 0.65 (±0.026) × K intake	45	12.9	3.55	3.57	92.88	0.28	0.96	0.97
= 195.16 (±54.83) + 2.32 (±28.62) × dietary K	45	49.4	0.05	26.21	73.74	1.09	−0.15	0.00
= 55.61 (±44.77) + 1.96 (±0.493) × water intake	18	41.4	0.17	0.70	99.13	0.75	0.60	0.66
= −35.79 (±24.11) + 12.16 (±0.785) × urine volume	26	39.5	19.63	3.50	76.87	0.71	0.72	0.80
= 214.33 (±241.33) + 0.501 (±7.38) × milk yield	20	89.6	4.06	84.87	11.08	1.51	−0.69	0.00
K_Fa_ (g/cow per day)								
= −3.32 (±7.90) + 2.73 (±0.391) × DMI	54	46.6	1.41	13.10	85.49	0.97	0.42	0.48
= 6.93 (±6.31) + 0.126 (±0.019) × K intake	54	41.3	0.17	7.38	92.45	0.86	0.54	0.59
= 28.74 (±12.45) + 6.85 (±7.15) × dietary K	49	49.3	0.57	6.48	92.95	1.03	−0.02	0.04
= 30.70 (±28.22) + 0.668 (±0.909) × milk yield	37	56.2	0.25	57.42	42.32	1.24	−0.38	0.05

1 K_Ur_ = urinary K excretion model; K_Fa_ = fecal K excretion model; dietary K = dietary K concentration; n = number of treatment means; RMSPE = root mean square prediction error, expressed as the percentage of the observed mean K excretion (g/d); MB = mean bias, expressed as the percentage of the total mean square prediction error; SB = slope bias, expressed as the percentage of the total mean square prediction error; ED = error due to random sources as the percentage of the total mean square prediction error; CCC = concordance correlation coefficient. Figure 1 demonstrates the performance of the proposed urinary and fecal K excretion models.

2 In parentheses: ±SE.

The performance metrics included Lin's concordance correlation coefficient (**CCC**) (Lin, 1989) calculated with the epiR package (Stevenson et al., 2022), total mean square prediction (**MSPE**) based on Equation 2 from Bibby and Toutenburg (1977), and root mean squared prediction error (**RMSPE**) expressed as a percentage of the observed mean (g/d). To identify systematic bias, the MSPE (Equation 2) was decomposed into mean bias (**MB**, Equation 3), slope bias (**SB**, Equation 4), and error due to random sources (**ED**, Equation 5). This decomposition enables the detection of whether the model exhibited a constant degree of overprediction (MB) or increasing overprediction tendencies with the predicted values (MB and SB) (Alvarez-Fuentes et al., 2016). These biases were expressed as a percentage of total MSPE.

[2] $$MSPE=\frac{\sum_{i=1}^{n}\;{\left(y_{i}-{\hat{y}}_{i}\right)}^{2}}{n},$$

where *y_i_* is the observed value of the urinary or fecal K excretion variables for the *i*th observation and *ŷ_i_* is the predicted value of the urinary or fecal K excretion variables for the *i*th observation.

[3] $$MB={\left(\bar{P}-\bar{O}\right)}^{2},$$

[4] $$SB={\left(S_{p}-r\times S_{o}\right)}^{2},$$

[5] $$ED\;=\left(1-r^{2}\right)\times {S_{o}}^{2},$$

where $\bar{P}$ and $\bar{O}$ are the predicted and observed means, respectively; *S_p_* is the predicted standard deviation, *S_o_* is the observed standard deviation, and *r* is the Pearson correlation coefficient.

The performance of the proposed models was ranked based on the lowest RMSPE and highest CCC values. The comparison of the cross-validated results of the proposed models was selected based on the lowest **RSR**, which is the ratio of RMSPE to the observed values' SD. This metric allows for a fair comparison of model performance, considering the number of observations used (Moriasi et al., 2007). In the current study, previously published equations were not evaluated because their measurements or observations were used in the development of the proposed models due to limited data in the literature.

The summary statistics and description of the urinary and fecal K excretion databases for the variables used to develop the urinary and fecal K (K_Ur_ and K_Fa_) excretion predictive models in our study are in Table 1. In the urinary database, the daily K excreted ranged from 70.2 to 476.0 g, with a mean (± SD) of 202.5 ± 91.1 g. In contrast, earlier studies reported lower values for nonlactating and lactating cows (Nennich et al., 2006; Kume et al., 2008). For instance, Kume et al. (2008) found a lower average value of 185.6 ± 106.0 g/d with a range of 32.5 to 438.8 g/d from nonlactating and lactating cows. The mean milk yields in the urinary and fecal K databases were 31.2 ± 6.2 and 30.0 ± 7.8 kg/d, respectively. In the current study, K intake varied from 105.3 to 686.0 g/d averaging 316.6 ± 144.3 g/d, whereas the DMI ranged from 4.4 to 27.6 kg/d with an average of 16.9 ± 5.4 kg/d. Greater variability was observed in K intake than in DMI (CV = 45.0% vs. 32.0%). This variability might be attributed to differences in forage source (Kume et al., 2004) and animal-related factors (e.g., stage of lactation and BW) (Silanikove et al., 1997; Jenkins et al., 2017). In addition to differences in DMI among studies, another source of variation could be due to dietary K content as dietary K content ranged from 0.79% to 2.73% of DM, with an average of 1.65% of DM. This is less than the reported values by Kojima et al. (2005) for dry and lactating cows, which were 2.33% and 1.96% of DM, respectively. In the present analysis, the daily urine volume was greater (range 10.5 to 39.9 kg/d) than that found by Kume et al. (2008) from 50 lactating cows ranging from 3.2 to 33.2 kg/d, and our results could have been influenced by a wide range of water intake (20.9 to 146.6 kg/d). In the fecal K excretion database, daily fecal K excretion, DMI, and K intake ranged from 15.5 to 94.7 g/d, 4.4 to 27.6 kg/d, and 105.3 to 583.0 g/d, respectively. These analyses revealed that dairy cattle in the dataset excreted 64.0% of consumed K via urine and 15.4% through feces, which are not surprising because it has been shown that urine serves as the primary excretory pathway for K in dairy cows (Lomba et al., 1969), whereas unabsorbed K is eliminated through feces (NRC, 2001).

The proposed urinary and fecal (K_Ur_ and K_Fa_) K excretion prediction equations are in Table 2. Due to a lack of improvement with increasing model complexity, simple linear regression equations were developed. Furthermore, our study was unable to establish predictive models for nonlactating and lactating cows due to the limited number of observations available and this approach differs from the experimental study of Kojima et al. (2005). Therefore, the current models can be generalized to lactating and nonlactating cows.

Dry matter and K intake had a positive relationship with K_Ur_ or K_Fa_ excretion. In addition, urine volume and water intake were positively related to K_Ur_ excretion. This corroborates with the study by Kume et al. (2008); however, there was no obvious relationship (*P* > 0.05) between dietary K content and either K_Ur_ or K_Fa_, suggesting that it is more appropriate to consider total K intake as a predictor than dietary concentration.

The model incorporating DMI predicted K_Ur_ with slightly less RMSPE and RSR of 44.6% and 0.98, respectively (Table 2) compared with K_Fa_. This model showed better predictions on the K_Fa_ excretion with a CCC of 0.42 and a greater coefficient of determination. However, this model showed significant slope biases for K_Fa_ (*P* < 0.05; Figure 1). The model including the milk yield showed poor predictive performance for both K_Ur_ and K_Fa_ with CCC of −0.70 and −0.38, respectively (Figure 1). This is supported by the study of Kojima et al. (2005), which did not identify milk yield as the predictor of either K_Ur_ or K_Fa_. Dietary K content and milk yield did not significantly predict K_Ur_ and K_Fa_ and this is evident by the poor performance (Figure 1) of the models including this variable, with the highest RSR >1 and lowest CCC <1. Alternatively, the model including water intake predicted K_Ur_ with negligible systematic biases (Figure 1) and a random bias of 99.1%. Water intake explained 66% of the observed variation in K_Ur_. Improvement in the K_Ur_ predictions was observed with urine volume compared with the model that included water intake (RSR: 0.71 vs. 0.75; CCC: 0.72 vs. 0.60); this model showed significant MB (*P* < 0.05). These results suggest that urine volume provides a more accurate reflection of the amount of K being excreted by the kidneys, making it a better predictor of urinary K excretion in cows than water intake. Feces is not a significant pathway for K excretion in ruminants accounting for only approximately 13% of the total K excretion (Ward, 1966). Urine volume is primarily influenced by the impact of K on urine osmolarity (Bannink et al., 1999). Consistent with the literature (Kume et al., 2008), K_Ur_ was positively correlated with urine volume. These results suggest that when urine volume increases, it allows for a greater elimination of K from the body via clearance in the kidneys.

**Figure 1:**
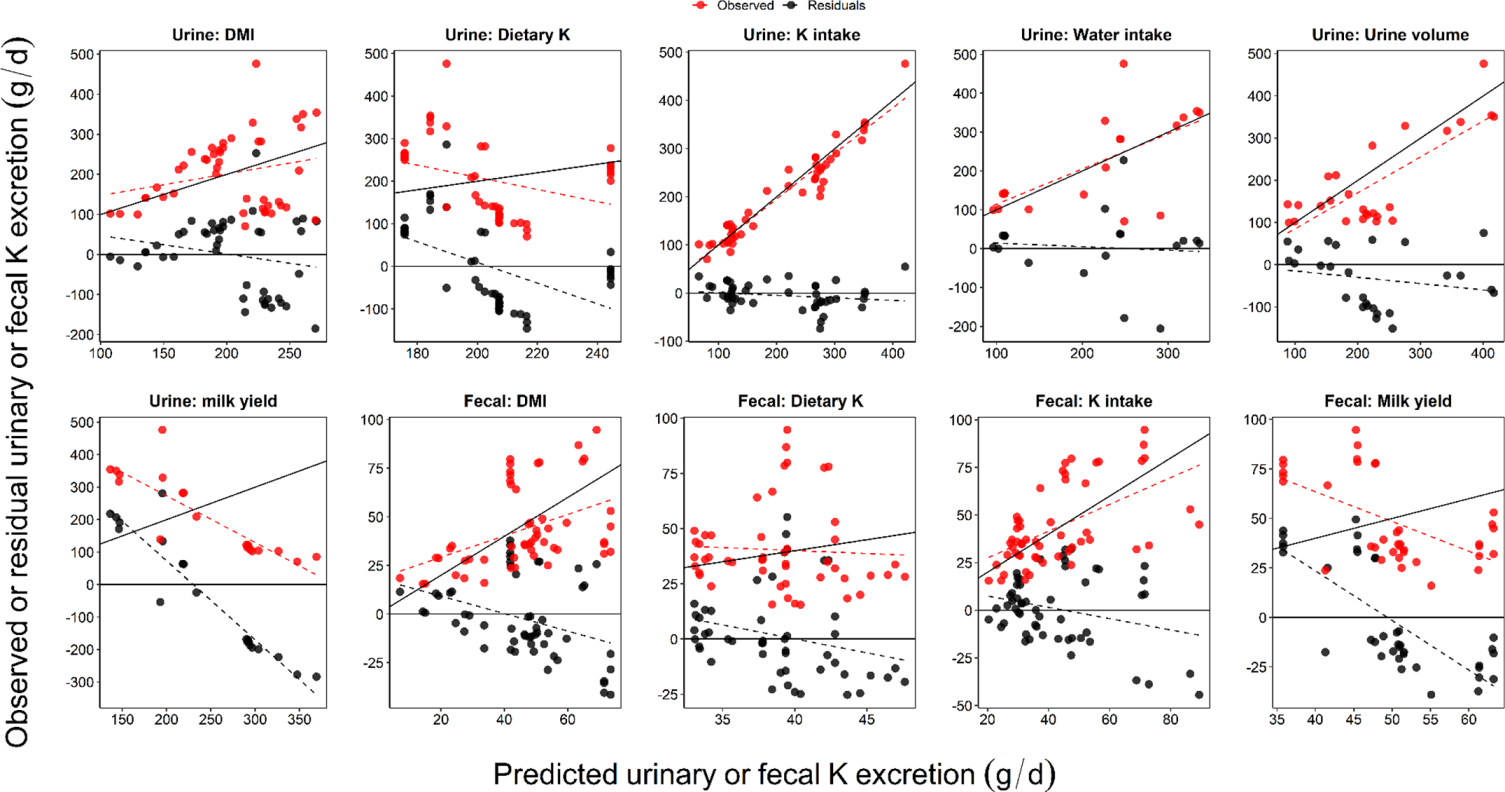
Plots of observed versus predicted urinary or fecal K excretion (g/d; red circles), and residuals (black circles: observed − predicted values) versus predicted K excretion (g/d; red circles) generated from urinary and fecal databases. The dashed red and black lines indicate the relationship between predicted and observed urinary or fecal K excretion and predicted values and the residuals. The solid black lines represent the identity line, where y = x (1:1).

Overall, this analysis indicates that K_Ur_ and K_Fa_ could be estimated using the amount of K consumed, accounting for 97% and 59% of observed variations in K_Ur_ and K_Fa_, respectively. Similar to these findings, earlier studies have predicted K_Ur_ or K_Fa_ from ingested K in lactating cows (Khorasani et al., 1997; Bannink et al., 1999; Kojima et al., 2005); however, these studies reported inconsistent and lower coefficients of determination (marginal R^2^) for predicted K_Fa_ compared with the current study. For instance, Khorasani et al. (1997) reported an R^2^ of 0.27 for the K_Fa_ equation as a function of K intake and the quadratic term of K intake, whereas Kojima et al. (2005) observed an R^2^ of 0.13 and 0.00 for the dry and lactating cows, respectively.

Alternatively, Kojima et al. (2005) found that K intake was responsible for 86% and 87% of observed variations in K_Ur_ for dry and lactating cows, respectively. The observed differences in the proposed K_Ur_ and K_Fa_ models, which incorporate K intake, and the published models are likely because the published models were generated from measurements taken under controlled laboratory experiments, where researchers can manipulate and control external sources of variation. In contrast, observational studies such as the current study use data from a broader range of treatments, DMI, and forages, which result in the greater variability observed in K intake. The greater R^2^ in our study is likely due to the weighting by the number of observations in the models' development to account for the variation among treatment means.

These results demonstrate that lowering K intake in lactating dairy cows will lower K excretion either through urine or feces. However, the proposed models in this study were based on data from cows with milk yields ranging from 30 to 41 kg/d. Consequently, the performance of these models for cows outside of this range remains uncertain, and further research is warranted with higher producing cows in a variety of environmental conditions. The proposed models should be used with caution due to inherent discrepancies from the limited number of observations, resulting in challenges encountered in constructing models for subcategories (such as lactating cows, nonlactacting cows, and heifers). Therefore, further research is encouraged to improve model development in this area by gathering and publishing additional data, including both individual animal and group data.

The simple regression models for both urinary and fecal K excretion developed in this study showed small systematic biases and these proposed models could be incorporated into decision-making tools to assist farmers and nutritionists to make informed decisions when formulating diets to optimize productivity while reducing the environmental impact of milk production.
